# Where do we look for the next breakthrough in sleep research?

**DOI:** 10.1186/s12883-023-03330-3

**Published:** 2023-07-28

**Authors:** Alessandro Viganò, Majid Ghareghani, Birendra Mallick, Simone Russo

**Affiliations:** 1 IRCCS - Fondazione Don Carlo Gnocchi, Milan, Italy; 2 Neuroscience Laboratory, CHU de Québec Research Centre, Department of Molecular Medicine, Faculty of Medicine, Laval University, Québec City, Québec G1V 4G2 Canada; 3 Amity Institute of Neuropsychology & Neurosciences, Amity University Campus, Noida, India; 4 Department of Biomedical and Clinical Sciences “L. Sacco”, University of Milan, Milan, Italy; 5 Department of Philosophy ‘Piero Martinetti’, University of Milan, Milan, Italy; 6 Wallace H Coulter Department of Biomedical Engineering, Georgia Institute of Technology, Atlanta, GA US

## Abstract

The wakefulness-sleep cycle sets the pace of our life. Sleep research examines the transition between wakefulness and sleep, its hormonal regulation, and its pathological disruption. Understanding sleep mechanisms would improve quality-of-life well beyond sleep itself. To this aim, we invite contributions for the Collection “sleep physiology and circadian rhythms”.

## Main text

Sleep is a fundamental biological function, and we humans spend about one third of our lifespan asleep. While historically sleep and wakefulness were seen as counterposed dichotomies, in the last few decades researchers have demonstrated that they reflect a *continuum*, spanning from high arousal to deep sleep. This *continuum* has been differentiated in multiple sub-stages through behavior and electrophysiology, traditionally leveraging on the combination of electroencephalogram (EEG) and electromyogram, and more recently transcranial magnetic stimulation (TMS/TMS-EEG) and intracranial EEG.

The wakefulness-sleep cycle emerges from a delicate interplay between our inner circadian cycle, set by brainstem and hypothalamic neurons, and outer environmental factors. The integration between our inner clock and peripheral inputs relies on key neural and body structures that orchestrate the complex framework of neuronal, endocrinal, and visceral events underpinning our behavioral state. This delicate balance can be disrupted by multiple factors, including network alterations, brain lesions, degenerative disorders, and neuroendocrine dysfunction. These disrupting factors can undermine brain energy resources, metabolism and functionality, thus affecting our performance and impacting everyday life.

Under physiological conditions, the wakefulness-sleep cycle adjusts neuronal synapses on a circadian basis [1]. During wakefulness, learning mechanisms promote synaptic potentiation by enhancing pre-existing synapses and generating new ones. In principle, this mechanism would constantly potentiate synapses throughout wakefulness, to the point of resulting in a hyperconnected network. To prevent this dysregulated connectivity, synaptic potentiation is self-limited by the synaptic pressure mechanism. As wakefulness persists, this mechanism progressively induces sleepiness and the intrusion of sleep-like slow waves in the awake brain [2], promoting sleep. By inducing selective synaptic downscaling, sleep renormalizes network connectivity while preserving important synapses and discarding unused synapses [3]. As such, sleep plays a key role in learning and memory consolidation [4].

To date, the regulation of the wakefulness-sleep cycle is a key subject area in sleep research. In particular, one of the most studied sleep regulators is melatonin, a hormone produced by the pineal gland in response to darkness. Melatonin contributes to sleep physiology by opening the thalamic “sleep gate” for non-rapid eye movement (NREM) sleep, significantly increasing sleep propensity at night [5] and improving REM sleep continuity [6] with beneficial effects on the circadian rhythm. By having the most physiological mechanism of action among sleep promoting drugs, it is not surprising that melatonin preparations are commonly used as a supplemental sleep aid and its FDA-approved receptor agonists are recommended for the treatment of many sleep disorders, including insomnia, by several sleep guidelines [7].

For this reason, the use of over-the-counter melatonin supplements for sleep disorders has risen dramatically in many countries: the global size of melatonin supplement market showed a two-fold increase between 2016 and 2021, and Ghareghani et al. [8] reported a seven-fold increase in melatonin consumption among children between 2007 and 2012. Although melatonin is broadly used, it is a hormone that has significant dose- and time-dependent effects on the body’s functions. Most recently, the first long-term follow-up study on melatonin consumers showed that 75% of melatonin consumers reported psychopathological problems, particularly anxiety disorder, within two years of their first prescription [9]. This work highlighted the importance of long-term studies to understand melatonin’s function in sleep physiology, as well as its potential side-effects. The field of sleep physiology would benefit from a better understanding of melatonin and its impact on human health.

An interesting touch point between sleep physiology and pathology is local sleep. Physiologically, local sleep occurs in animals and some evidence indicates that it also emerges in humans after prolonged wakefulness [10]. Pathologically, local sleep is described after several types of brain injury, such as trauma, anoxia and stroke, and it can contribute to patients’ symptoms and disability. The complex clinical presentation of these disorders may derive from the induction of local sleep during wakefulness in intact brain areas close and connected to the lesion, potentially reflecting network dysfunctions [11].

The exact role of these sleep-like slow waves is still under investigation, particularly in understanding whether this sleep-like activity represents a dormancy state or a plastic remodeling of altered networks. Indeed, areas exhibiting sleep-like activity become incapable of sustaining high complexity states and the electrophysiological recovery of these areas parallels and, in some cases, anticipates patients’ clinical improvement [12, 13]. This is quite puzzling because slow wave activity in a strokes’ proximity, such as the cortical spreading depolarization observed during massive brain infarcts and hemorrhages, is generally associated with poorer outcomes and may represent an extreme form of cortical spreading depression (CSD) [14]. On the other hand, in a murine model, triggering CSD causes an increase in NREM sleep in the same brain areas during subsequent sleep time [15], suggesting plasticity. Overall, there is a paucity of research on the relationship between CSD and sleep in humans.

The last decades have therefore been a turning point in our understanding of many aspects of sleep physiology and pathology. Sleep research has gone from being a niche topic to becoming a top-tier research field, and there is clear public interest for further advances to be implemented in our everyday lives. For this reason, we hope that this Collection will foster innovative and promising research papers to consolidate the link between the fundamental mechanisms of sleep and diseases, laying the bases for innovative approaches to disease prevention, diagnosis, prognosis, and treatment, further extending the boundaries of sleep research.

## Data Availability

Not applicable.

## Abbreviations

EEG: electroencephalogram
TMS/TMS-EEG: transcranial magnetic stimulation
NREM: non-rapid eye movement
REM: rapid eye movement
CSD: cortical spreading depression
